# Wenckebach responsive to cephalosporins – it must be Lyme carditis

**DOI:** 10.3402/jchimp.v3i3-4.23013

**Published:** 2013-12-17

**Authors:** Lauren B. Dobbs, Marc A. Mugmon

**Affiliations:** 1 Department of Medicine, Greater Baltimore Medical Center, Towson, MD, USA; 2 Union Memorial Medical Center, Baltimore, MD, USA

**Keywords:** Lyme carditis, Wenckebach, Mobitz 1, AV block

## Abstract

A 37-year-old female presented to the hospital with erythema migrans and fatigue developed hypotension and variable episodes of AV block, including both Mobitz I and complete AV block. She was treated with IV antibiotics and her arrhythmia resolved within 24 hours without any further intervention.

The patient was a 37-year-old previously healthy mother of five who presented to the ED with progressive fatigue, headache, an erythematous macular rash with central clearing, as well as new onset numbness and tingling of her face. She stated that she had become progressively fatigued to the point that at the same time last year she was an avid runner, and now hardly had the energy to walk around the block. In the month previous, she had traveled to the beaches of NC with her family, and had developed a febrile illness upon her return with a fever up to 103°F. She then developed a persistent rash, which fluctuated both in size and location. These lesions were fairly large in diameter and irregular in shape. They were erythematous, but not painful or pruritic and were found on her anterior and posterior thorax, as well as her abdomen and lower extremities. She lived on a farm in a rural region of Maryland and had seen ticks on her children and dogs although she did not recall seeing one on her person. Her son had been recently diagnosed with Lyme disease and treated with antibiotics although whether he was formally tested was unclear.

On admission, she underwent a lumbar puncture with cerebrospinal fluid analysis which was found not to be indicative of bacterial meningitis with a glucose of 59, protein 39, no WBC, and a small number of RBCs which decreased from 18,100 in the first tube to 100 in the second tube, thought to be from a traumatic tap. Her labs were otherwise insignificant with a normal WBC count, mild anemia, and an unremarkable complete metabolic panel and UA. Physical examination was remarkable only for the rashes described above. An initial EKG performed in the ED revealed normal sinus rhythm.

She was admitted to the telemetry unit and started on IV ceftriaxone at 2 g every 12 hours for the treatment of suspected disseminated Lyme disease. A few hours after admission, she developed hypotension and her telemetry readings started showing episodes of AV block. Multiple EKGs were taken during this time, which revealed periods of preserved conduction as well as episodes of Mobitz I with 4:3 AV conduction, 3:2 AV conduction, 2:1 AV conduction, and some episodes of complete AV block with a junctional escape rhythm (Figs. 1 and 2).

**Fig. 1:**
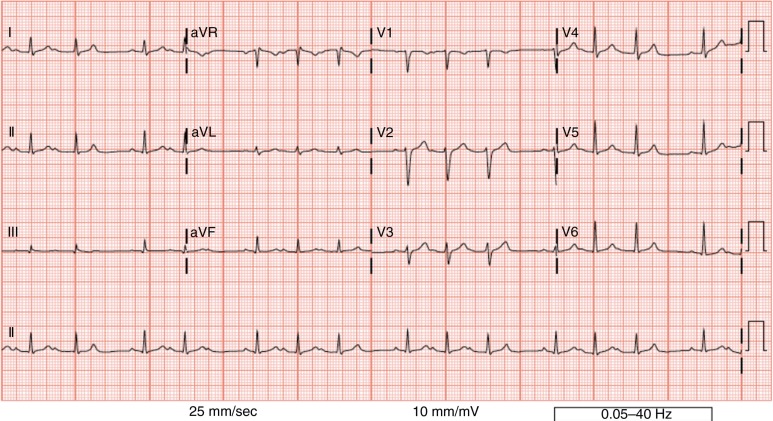
This tracing demonstrates Mobitz 1 (Wenckebach) with variable conduction, primarily 4:3 conduction ratios.

**Fig. 2:**
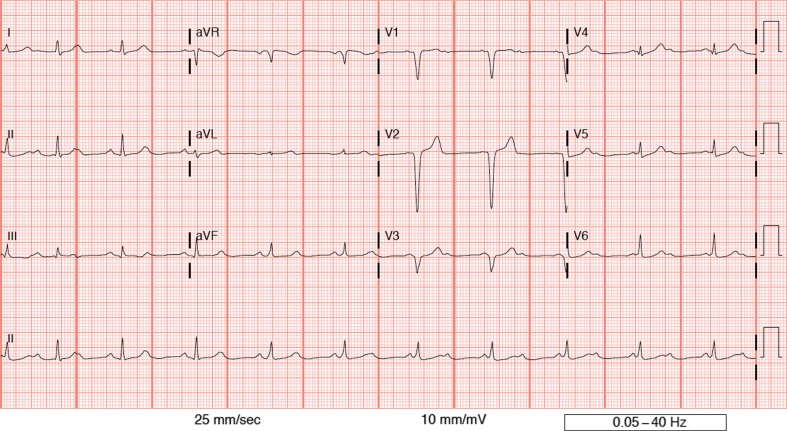
This tracing demonstrates sinus tachycardia and impaired AV conduction. Initially, Mobitz I is likely, followed by complete AV block and a junctional escape rhythm at a rate of 64.

She remained asymptomatic throughout these episodes without syncope, dyspnea, or diaphoresis. No cardiac intervention was performed, and within 24 hours she had returned to normal sinus rhythm, and remained in normal sinus rhythm for the duration of her hospital stay with antibiotic treatment alone. A Lyme antibody screen was found to be positive, as well as a Lyme Western blot, which was positive for IgM but negative for IgG. She was discharged from the hospital with a peripherally inserted central catheter line and a 4-week course of IV ceftriaxone with follow-up planned with an infectious disease specialist.

## Discussion

In August 2013, the Centers for Disease Control (CDC) released a new estimate that approximately 300,000 Americans develop Lyme disease each year, despite the fact that only 30,000 are reported to the CDC (1). Of those who go untreated, studies have determined that 4–10% will develop Lyme carditis, which usually starts 3–5 weeks after the rash is evident (2). Although Lyme disease classically presents with erythema migrans and joint pain, it is possible that these will be absent and carditis can be the only presenting symptom (2). Lyme carditis most often manifests itself as atrioventricular conduction disturbances, but myocarditis, left ventricular dysfunction, cardiomegaly, and pericarditis are also possible (3). Although rare, reports of chronic dilated cardiomyopathy and other conduction disturbances have been described (3,4).

The AV block of Lyme carditis tends to fluctuate and may quickly progress from first degree block to second degree or complete heart block within minutes. In two separate reviews of patients with Lyme carditis, 49–54% had complete AV block (4). As with other forms of AV block, those with higher degrees of block were more likely to be symptomatic with syncope, dizziness, palpitations, and dyspnea (4). Currently, the indications for pacing in these patients are the same as for other causes of AV block. Patients who are symptomatic due to severe AV block may require placement of a temporary pacemaker (4). It has been reported that AV block secondary to Lyme disease usually lasts 3–42 days (4) with complete heart block resolving usually within 1 week, although in this case the documented AV block lasted less than 24 hours (4). The overall prognosis is good given that the carditis typically resolves with antibiotic treatment alone, and cardiac intervention is often unnecessary (4).
